# Supplementary material for: m5CRegpred: Epitranscriptome Target Prediction of 5-Methylcytosine (m5C) Regulators Based on Sequencing Features
Source: Genes (Basel). 2022 Apr 12;13(4):677. doi: 10.3390/genes13040677 (PMC9025882; doi:10.3390/genes13040677)
Supplement: Supplementary file 1 [file genes-13-00677-s001.zip › Supplement Tables S1 and S2.pdf]

Table S1 Cross-validation with different features

	Mature mRNA model			Full transcript model			Average
	NSUN2	YBX1	ALYREF	NSUN2	YBX1	ALYREF	
EIIP	0.711	0.759	0.809	0.680	0.669	0.686	0.719
autoCor	0.509	0.601	0.603	0.480	0.499	0.604	0.549
crossCor	0.561	0.647	0.650	0.554	0.515	0.515	0.574
PseKNC	0.649	0.746	0.767	0.623	0.649	0.657	0.682
ChemProper	0.602	0.718	0.748	0.575	0.645	0.704	0.665
ONE_HOT	0.623	0.718	0.740	0.595	0.641	0.693	0.668
CONPOSI	0.712	0.771	0.826	0.717	0.668	0.793	0.748
PSNP	0.858	0.787	0.849	0.849	0.763	0.907	0.835

Table S2 Independent test with two features combination

	Mature mRNA model			Full transcript model		
	NSUN2	YBX1	ALYREF	NSUN2	YBX1	ALYREF
EIIP_autoCovar	0.647	0.728	0.841	0.719	0.768	0.841
EIIP_crossCovar	0.659	0.716	0.847	0.726	0.756	0.847
EIIP_PseKNC	0.657	0.725	0.848	0.722	0.765	0.848
EIIP_ChemicalProper	0.602	0.650	0.778	0.706	0.69	0.778
EIIP_ONE_HOT	0.603	0.649	0.777	0.703	0.689	0.777
EIIP_CONPOSI	0.656	0.724	0.849	0.721	0.764	0.849
autoCovar_crossCovar	0.619	0.606	0.713	0.634	0.646	0.713
autoCovar_PseKNC	0.676	0.691	0.749	0.689	0.731	0.749
autoCovar_ChemicalProper	0.605	0.651	0.778	0.700	0.691	0.778
autoCovar_ONE_HOT	0.604	0.650	0.778	0.706	0.690	0.778
autoCovar_CONPOSI	0.647	0.728	0.841	0.719	0.768	0.841
crossCovar_PseKNC	0.675	0.679	0.792	0.766	0.719	0.792
crossCovar_ChemicalProper	0.605	0.651	0.778	0.700	0.691	0.778
crossCovar_ONE_HOT	0.604	0.650	0.778	0.706	0.690	0.778
crossCovar_CONPOSI	0.659	0.716	0.847	0.726	0.756	0.847
PseKNC_ChemicalProper	0.660	0.695	0.801	0.772	0.735	0.801
PseKNC_ONE_HOT	0.654	0.694	0.798	0.766	0.734	0.798
PseKNC_CONPOSI	0.657	0.725	0.848	0.722	0.765	0.848
ChemicalProper_ONE_HOT	0.606	0.651	0.778	0.701	0.691	0.778
ChemicalProper_CONPOSI	0.657	0.715	0.835	0.728	0.755	0.835
ONE_HOT_CONPOSI	0.654	0.715	0.836	0.730	0.755	0.836
